# High-Performance Differential Imaging via Reconfigurable Black Phosphorus p–n Homojunction Optoelectronics

**DOI:** 10.1007/s40820-026-02104-z

**Published:** 2026-02-28

**Authors:** Rui Hao, Lili Luo, Lu Yang, Xue Yang, Fengsong Gao, Meijie Zhu, Yingtao Li, Qingliang Feng, Zemin Zhang

**Affiliations:** 1 School of Physical Science and Technology, Lanzhou University, Lanzhou, 730000 People’s Republic of China; 2 School of Chemistry and Chemical Engineering, Northwestern Polytechnical University, Xi’an, 710072 People’s Republic of China

**Keywords:** Ferroelectric, Reconfigurable, Black phosphorus, BiFeO_3_, Differential imaging

## Abstract

The advancement of differential imaging and adaptive machine vision demands hardware capable of dynamic signal modulation, yet traditional photodetectors are limited by static doping profiles and fixed junction polarities. To overcome this bottleneck, we present a reconfigurable black phosphorus (BP) p–n homojunction photodetector engineered via in situ ferroelectric domain programming. By leveraging the non-volatile ferroelectric field of a bismuth ferrite substrate, we achieve precise, nondestructive modulation of the BP band structure, allowing for reversible switching between p–n and n–p configurations within a single device channel. This ferroelectric doping strategy effectively eliminates interface damage associated with ion implantation while enabling programmable rectification behaviors. The device demonstrates self-powered operation with a responsivity of 44 mA W^−1^ at 808 nm under zero-bias conditions. Crucially, we demonstrate a single-pixel imaging prototype where the reconfigurable junction polarity enables tunable edge sharpness and high-fidelity image reconstruction. This work establishes a paradigm for ferroelectrically programmable 2D devices, providing a versatile platform for differential imaging and contrast-enhancement optoelectronic applications.

**Supplementary Information:**

The online version contains supplementary material available at 10.1007/s40820-026-02104-z.

## Highlights

- Ferroelectric BiFeO_3_ gating dynamically switches the black phosphorus (BP) homojunction between p–n and n–p states, enabling non-volatile reconfiguration.
- BP/BiFeO_3_ heterostructure achieves broadband self-powered photodetection from 365 to 1550 nm, with responsivity ranging from 44 mA W^–1^ (808 nm) to 1.6 A W^−1^ (425 nm) and specific detectivity up to 1.8×10^12^ Jones.
- Differential imaging leverages reversible p–n/n–p polarity switching to cancel common-mode static noise and enhance edge detection in near-infrared imaging.

**Supplementary Information:**

The online version contains supplementary material available at 10.1007/s40820-026-02104-z.

## Graphical abstract

**Figure:**
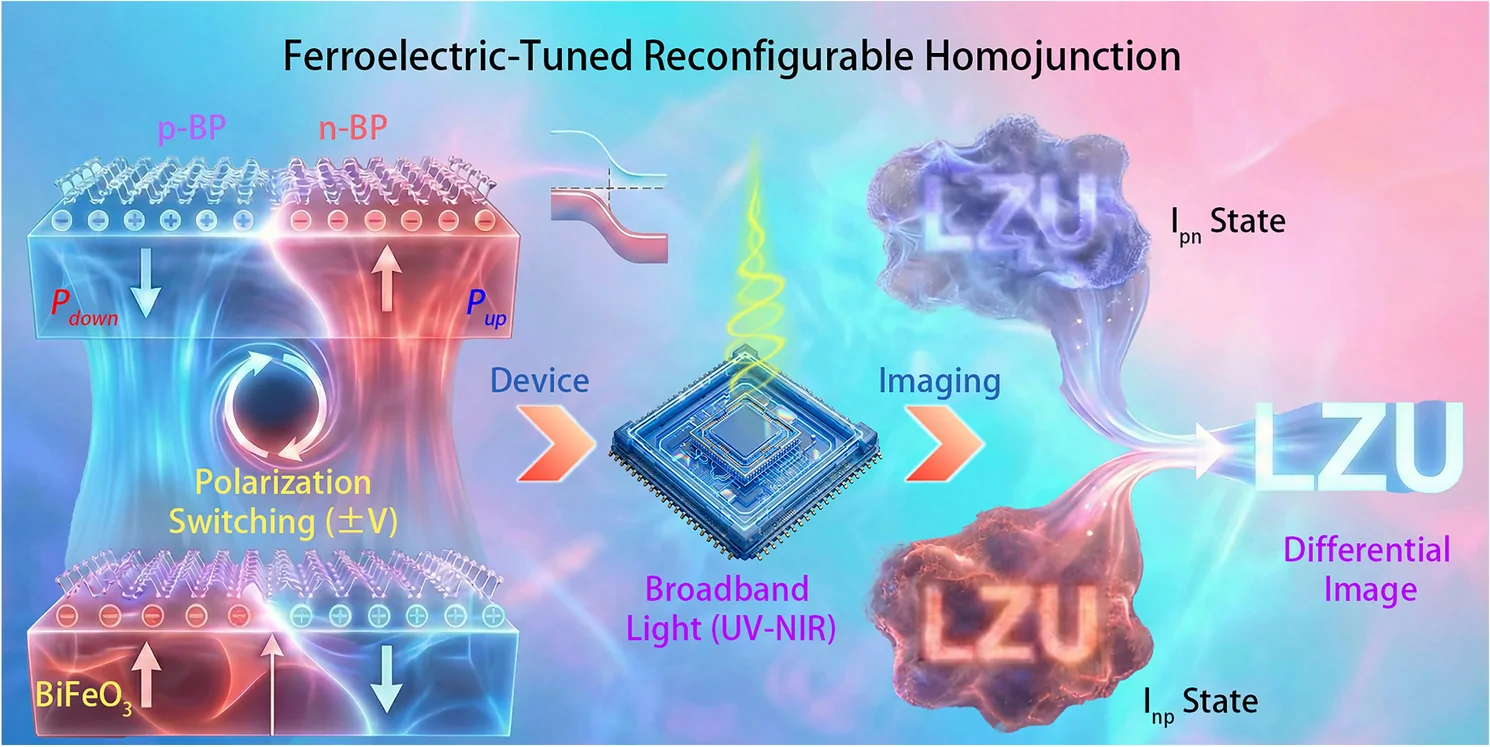

## Introduction

The shift toward intelligent machine vision requires hardware capable of “in-sensor” processing, moving beyond simple intensity detection to advanced differential imaging [1, 2]. To achieve this, optoelectronic devices must evolve from static architectures to reconfigurable systems capable of adapting to dynamic optical inputs [3–5]. Two-dimensional (2D) materials, particularly black phosphorus (BP), are uniquely suited for this challenge due to their tunable direct bandgap (0.3 ~ 2.0 eV) and high carrier mobility [6–9]. While conventional p–n junctions are static, the atomic thickness of BP allows its conductivity to be locally inverted via external fields, creating reconfigurable homojunctions within a single flake [10–12]. This ability to dynamically switch between p–n and n–p states unlocks programmable functionalities within a single device and enables sophisticated applications such as differential imaging.

However, realizing high-quality, stable, and precisely positioned p–n homojunctions in 2D materials, especially BP, remains a critical bottleneck [13]. Current doping technologies present a dilemma. "Hard" methods, such as ion implantation [14, 15] and plasma treatment [16], offer precise dopant profiling but inevitably introduce significant crystal lattice damage, which is particularly detrimental to atomically thin materials. On the other hand, "soft" strategies face their own challenges. Chemical doping via surface adsorption or substitutional doping is often unstable, non-uniform, or difficult to control [17]. Electrostatic gating, using split-gate architectures, can define clean p–n junctions, but this approach is volatile, requiring a constant power supply to maintain the doping state [18, 19].

Ferroelectric domain engineering offers a promising nondestructive pathway to induce stable, non-volatile electrostatic doping [19–22]. While recent advances utilizing organic polymers (e.g., P(VDF-TrFE)) [23] or periodically poled lithium niobate (PPLN) [24] have demonstrated the feasibility of ferroelectric-gated 2D devices, they face inherent limitations in reconfigurability and integration. Organic ferroelectrics often suffer from limited thermal stability and weaker polarization. PPLN typically relies on pre-patterned, fixed domain configurations in bulk crystals, which restricts the device to static operations. In contrast, bismuth ferrite (BFO) emerges as an ideal candidate for dynamically reconfigurable optoelectronics. BFO can be fabricated as high-quality epitaxial thin films, allowing for seamless integration with planar device architectures [25]. More importantly, beyond its colossal remnant polarization (up to 100 µC cm^−2^) and high Curie temperature (830 °C), BFO thin films allow for in situ, reversible domain switching via external voltages [26]. This unique capability enables the realization of truly programmable p–n homojunctions that can be dynamically modulated during operation, a feat not easily achievable with pre-poled bulk ferroelectrics.

In this work, we present a reconfigurable BP p–n homojunction photodetectors via BFO ferroelectric polarization for advancing differential imaging. The ferroelectric field generated by BFO induces pronounced band bending and Fermi-level modulation in BP, establishing a stable built-in electric field that enhances the separation and transport of photogenerated carriers. By reversibly switching the ferroelectric polarization direction, the BP channel can be dynamically programmed into either the p–n or n–p state. As a result, the BP homojunction exhibits clear rectifying characteristics and stable zero-bias operation, achieving a responsivity of 44 mA W^−1^ at 808 nm while maintaining broadband self-powered detection from 365 to 1550 nm. TCAD simulations further confirm the ferroelectric-field-induced band modulation and carrier redistribution. Crucially, a straightforward differential imaging approach that enables high-fidelity near-infrared image reconstruction with enhanced edge definition was achieved by alternating between the p–n and n–p states. This strategy offers a general route toward reconfigurable homojunctions and provides a useful platform for self-powered optoelectronic and imaging applications.

## Experimental Section

### Preparation of Polycrystal BP

BP bulk (20 mg, > 99.999%, MK NANO, www.mukenano.com) served as a cathode, while a Pt sheet was employed as an anode. The electrodes were set in parallel with a constant distance of 2.00 cm. Following previous reports [27, 28], tetra-*n*-butylammonium acetate (TBA·CH_3_COO) was dissolved in anhydrous, deoxygenated dimethylformamide to prepare the electrolyte, with a TBA·CH_3_COO concentration of 0.002 M. Electrochemical intercalation/exfoliation was carried out by applying a constant bias of 20.0 V for 15 min, after which the obtained BP was collected for subsequent transfer and device fabrication.

### Preparation of Polycrystal BFO Thin Films

Single-crystal BFO films were synthesized using pulsed laser deposition. A conductive (La,Sr)MnO_3_ (LSMO) buffer layer was initially placed on an atomically smooth (001) SrTiO_3_ (STO) substrate, succeeded by the epitaxial development of the BiFeO_3_ (BFO) layer. LSMO and BFO were synthesized in an oxygen environment at 700 °C, utilizing a base vacuum of 3 × 10^–3^ Pa, with oxygen pressures of 25 Pa for durations of 10 min, respectively. Throughout the deposition, the laser fluence was sustained at 1.2 J cm^−2^ with a repetition frequency of 5 Hz. After deposition, the samples were cooled down slowly to room temperature. The resultant BFO single-crystal film exhibited a thickness of around 50 nm.

### BP p–n Homojunction

The BP/BFO-based devices were fabricated using standard photolithography and magnetron sputtering techniques. Patterned Ti/Au electrodes with thicknesses of 20/50 nm were first deposited on the BFO substrate by photolithography, magnetron sputtering, and lift-off processes. Subsequently, few-layer BP flakes were precisely transferred onto the electrode-patterned BFO surface using a polydimethylsiloxane (PDMS)-assisted dry-transfer method.

### Electrical and Photoelectronic Characterization

The electronic characteristics of the device were assessed by using a KEITHLEY 2612B. The incident optical power at the sample plane was calibrated using a Thorlabs PM100A optical power meter. The responsivity was calculated as *R* = *I*_ph_/*P*_in_ (mA W^−1^). Since the beam spot fully covered the BP channel, the effective active area was defined as the BP channel area, *A* = 4 × 10^–6^ cm^2^. Optical images were taken on an Olympus BX51 microscope. AFM characterization was carried out with Bruker Multimode 8 system. Raman spectra were collected using a HORIBA RRANCE SAS-LabRAM Odyssey spectrometer with a 532 nm excitation laser. The laser power at the sample was set to 15 mW, the integration time was 10 s, and a 10 × objective was used for spectral acquisition.

## Results and Discussion

### Ferroelectric Engineered Homojunction

**Fig. 1:**
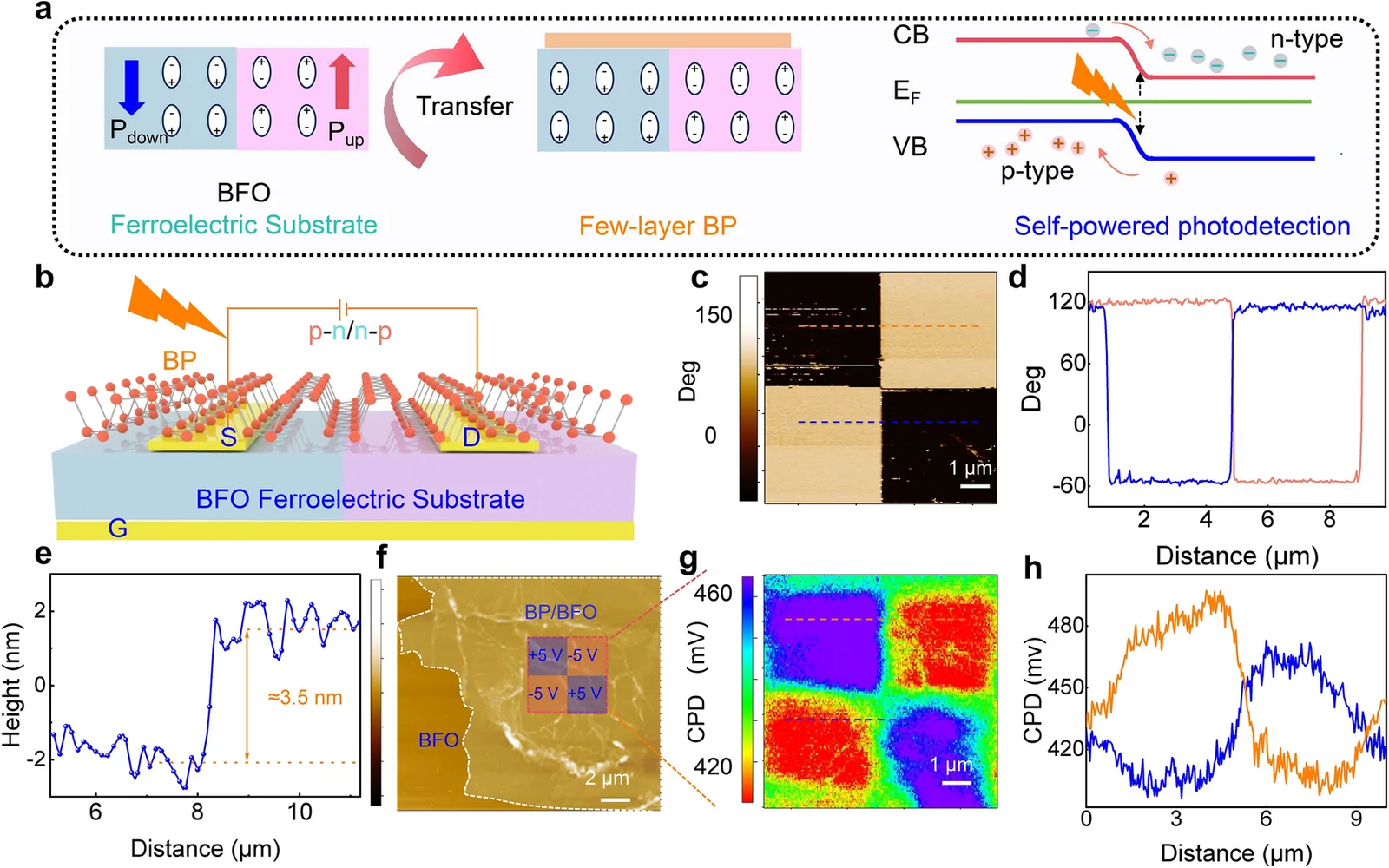
Ferroelectric Engineered Homojunction. Schematic illustration of **a** the few-layer BP p–n homojunction and **b** diagram of device structure. **c** PFM phase image of the BFO substrate under opposite polarization states and **d** corresponding phase profiles showing a 180° shift. **e–f** AFM thickness characterization of transferred BP (~ 3.5 nm, ~ 7 layers). **g** Extracted potential profiles and** h** JPFM mapping of polarized domains

The fabrication of a damage-free BP p–n homojunction photodetector relies on spatially modulating p- and n-type regions via the ferroelectric domains of a BFO substrate. These domains induce corresponding electron-deficient (p-type) and electron-rich (n-type) areas in the overlying few-layer BP, creating a built-in electric field that separates photogenerated carriers (Fig. 1a). The schematic structure of the few-layer BP/BFO heterojunction device is illustrated in Fig. 1b. High-quality BP nanosheets were first obtained via electrochemical exfoliation (Fig. S1) [27, 28], followed by a polydimethylsiloxane (PDMS)-assisted dry-transfer process to precisely place few-layer BP onto the BFO substrate with pre-patterned electrodes. The X-ray diffractions pattern confirms the epitaxial growth of a single-crystal BFO film with excellent phase purity (Fig. S2). Owing to the van der Waals nature of BP, no dangling bonds are present at the interface, resulting in a compact and stable heterointerface that facilitates the subsequent modulation of carrier transport in the BP channel by ferroelectric polarization. The polarization programming and characterization were carried out as schematically illustrated in Fig. S3a, b. Applying ± 5 V biases to the BFO layer reconfigures its ferroelectric polarization (*P*_up_/*P*_down_), enabling programmable p–n/n–p homojunctions within the same BP channel. Figure 1c presents the piezo-response force microscopy (PFM) phase image of the BFO substrate under different polarization fields. The phase profiles extracted along the orange and blue lines (Fig. 1d) reveal a phase difference of nearly 180° between adjacent domains, confirming the complete reversibility and opposite orientations of ferroelectric polarization in BFO. The atomic force microscopy (AFM) thickness profile (Fig. 1e) indicates a layer thickness of approximately 3.5 nm, corresponding to ~ 7 BP layers. This thickness range ensures both stable semiconducting behavior and relatively high carrier mobility.

To directly probe the ferroelectric polarization's effect, the surface potential of BP after applying a ± 5 V bias to patterned regions was mapped (Fig. 1f). The resulting Kelvin probe force microscopy (KPFM) image (Fig. 1g) shows a pronounced potential contrast between BP over *P*_down_ and *P*_up_ domains. Line profiles (Fig. 1h) confirm that the surface potential decreases by 45 ~ 65 mV over P_down_ regions relative to *P*_up_ regions. This reversible shift stems from electrostatic modulation by polarization-bound charges at the BP/BFO interface, enabling precise control over the BP channel's carrier density and band structure. Consequently, the BFO substrate creates a controllable surface potential gradient in few-layer BP, facilitating reconfigurable p–n/n–p homojunctions.

### In Situ Reconfiguration

**Fig. 2:**
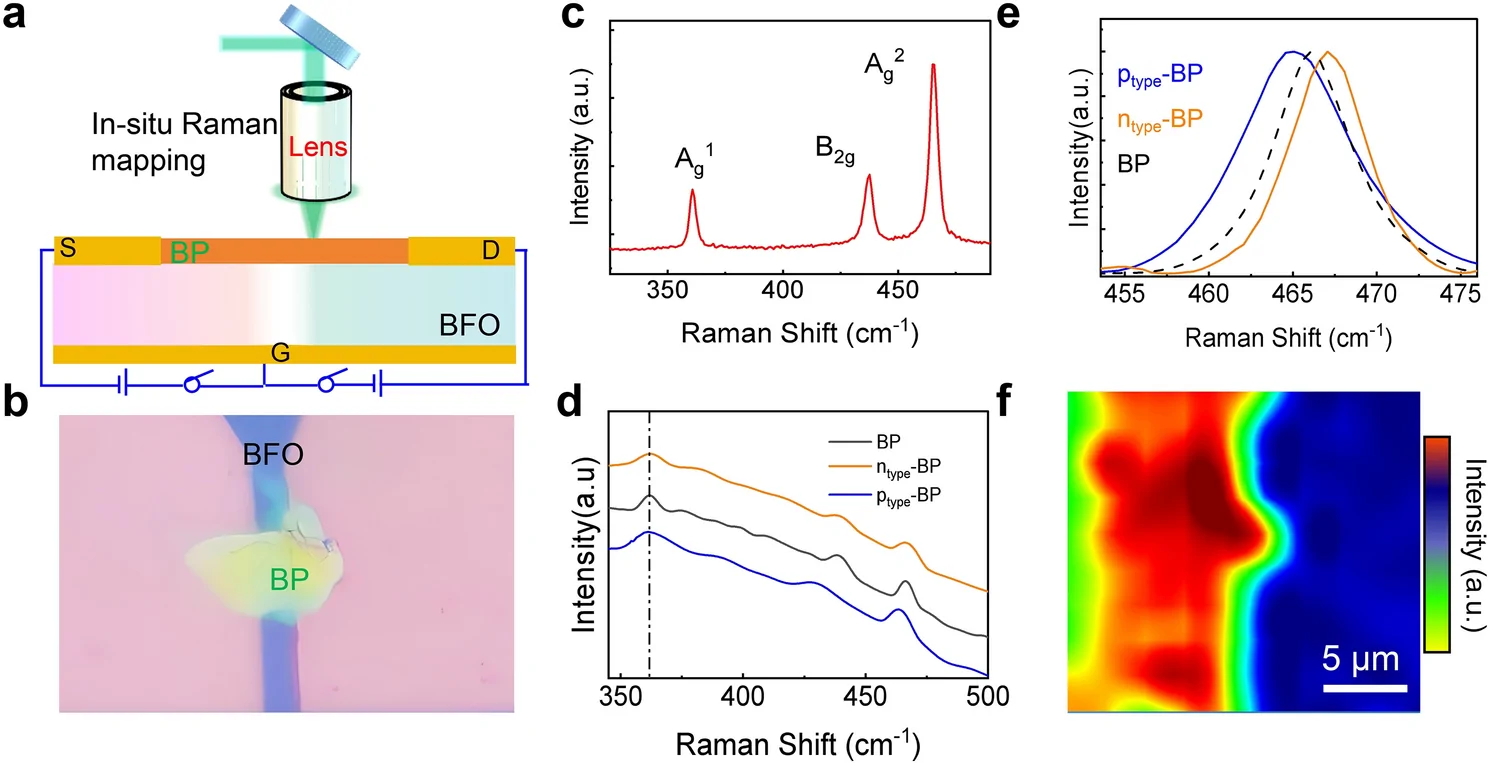
**a–b** In situ reconfiguration. Schematic illustration and optical image of the BP p–n homojunction used for in situ Raman characterization. Raman spectra of** c** BP and **d** BP on oppositely polarized BFO substrates. **e** Fine scanning of the BP A_g_^2^ characteristic peak and** f** the corresponding Raman intensity mapping over regions with opposite ferroelectric polarization

Raman spectroscopy directly probes the lattice alteration in BP induced by the BFO substrate's ferroelectric polarization (Fig. 2a). The polarization field from adjacent *P*_down_ and *P*_up_ domains modulates BP's electrostatic environment, redistributing carriers and strain and thus altering its phonon vibrations. The device optical image is shown in Fig. 2b. The Raman spectrum of pristine BP (Fig. 2c) shows three characteristic peaks: A_g_^1^ (362 cm^−1^), B_2g_ (439 cm^−1^), and A_g_^2^ (466 cm^−1^). Critically, all features of both BP and BFO (Eg and A_g_^1^ modes in Fig. S4a) are preserved in the heterojunction (Fig. S4b), confirming that the integration process is nondestructive and maintains lattice integrity.

To probe the effect of ferroelectric polarization on BP's vibrational properties, Raman peak shifts across three regions were analyzed: intrinsic BP (unpolarized), p-type BP (on* P*_down_), and n-type BP (on* P*_up_). Despite a slight modification of the spectral shape by the BFO substrate, the three main BP modes (A_g_^1^, B_2g_, A_g_^2^) remained distinct. Notably, the elevated baseline is attributed to the optical background from the BFO substrate, as confirmed by control Raman spectra measured on bare BFO and BP/BFO under identical conditions (Fig. S4). While the A_g_^1^ mode was stable, the A_g_^2^ mode exhibited significant polarization-dependent shifts (Fig. 2d). Fine scanning of the A_g_^2^ mode (~ 466 cm^−1^) revealed a pronounced redshift on the P_down_ domain and a blue-shift on the *P*_up_ domain. Single-point spectra from the domain centers (Fig. 2e) quantified these shifts at approximately − 1.44 and + 1.01 cm^−1^, respectively.

In situ Raman mapping of this mode (Fig. 2f) spatially confirms that ferroelectric polarization electrostatically modulates both carrier type and electron–phonon coupling in BP. The observed shifts stem from BP's strong electron–phonon coupling, where p-type doping softens the A_g_^2^ phonon (redshift) and n-type doping stiffens it (blue-shift). These results, consistent with prior reports [29, 30], demonstrate that the BFO substrate reversibly controls the local doping in BP, directly confirming the formation of reconfigurable homojunctions.

### Mechanism Simulations

**Fig. 3:**
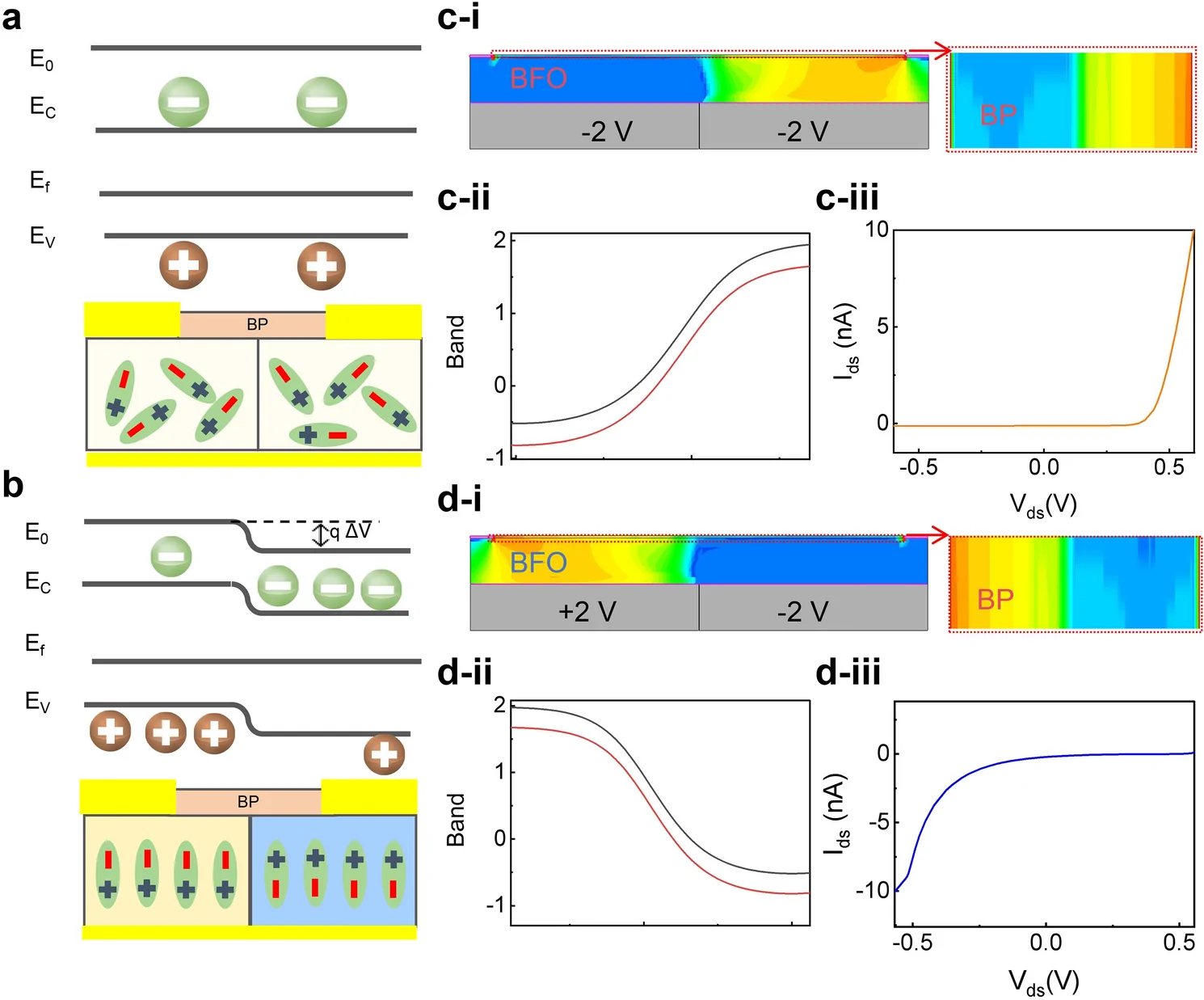
**a** Proposed mechanism of reconfigurable BP homojunctions. Nonpolarized BFO yields uniform carrier distribution, **b** while opposite ferroelectric domains induce electron–hole diffusion**. c-i, d-i** TCAD simulations of carrier distribution**, c-ii, d-ii** band diagrams, and **c-iii, d-iii** I–V characteristics under opposite polarizations

Above analyses establish that BFO's ferroelectric polarization reliably controls the carrier type and concentration in BP. The underlying physical mechanism, illustrated in Fig. 3a, b, involves direct electrostatic modulation of BP's band structure. On nonpolarized BFO, BP maintains a uniform carrier profile. When contacted with oppositely polarized *P*_down_ and *P*_up_ domains, holes and electrons accumulate in the overlying BP, respectively. The resulting concentration gradients drive bidirectional carrier diffusion, establishing a space-charge region and a built-in electric field at the domain wall. This field generates a drift current that eventually balances the diffusion current at electrostatic equilibrium, aligning the Fermi levels and creating a built-in potential. KPFM measurements confirm this potential to be ~ 65 mV, directly evidencing the p–n homojunction.

To further validate the influence of ferroelectric domain polarization on carrier distribution and band alignment in BP, TCAD simulations were carried out under two representative polarization configurations. Figure 3c–i and d–i presents the electron distribution profiles corresponding to the p–n and n–p configurations, respectively. The symmetric but complementary electron concentration profiles observed under opposite polarization states confirm the controllable and reversible formation of lateral p–n/n–p junctions. The corresponding band diagrams (Fig. 3c-ii and d-ii), obtained by self-consistently solving the Poisson equation, carrier continuity equations, and drift–diffusion model, clearly illustrate the modulation of junction polarity by ferroelectric polarization. When the applied voltage is switched from + 2/ − 2 V to − 2/ + 2 V, the Fermi level shifts relative to the conduction and valence band edges of the BP channel, leading to inversion of the built-in electric field direction and a continuous transition between p–n and n–p junction configurations. Notably, asymmetric shifts in the conduction band minimum (CBM) and valence band maximum (VBM) are observed on either side of the junction, indicative of a spatial redistribution of carriers and electrostatic potential. Finally, the simulated I–V characteristics (Fig. 3c-iii and d-iii) exhibit opposite rectification, matching our experimental data and conclusively demonstrating that BFO polarization enables stable, programmable control over BP's carrier polarity.

### Reconfigurable Optoelectrical Properties

**Fig. 4:**
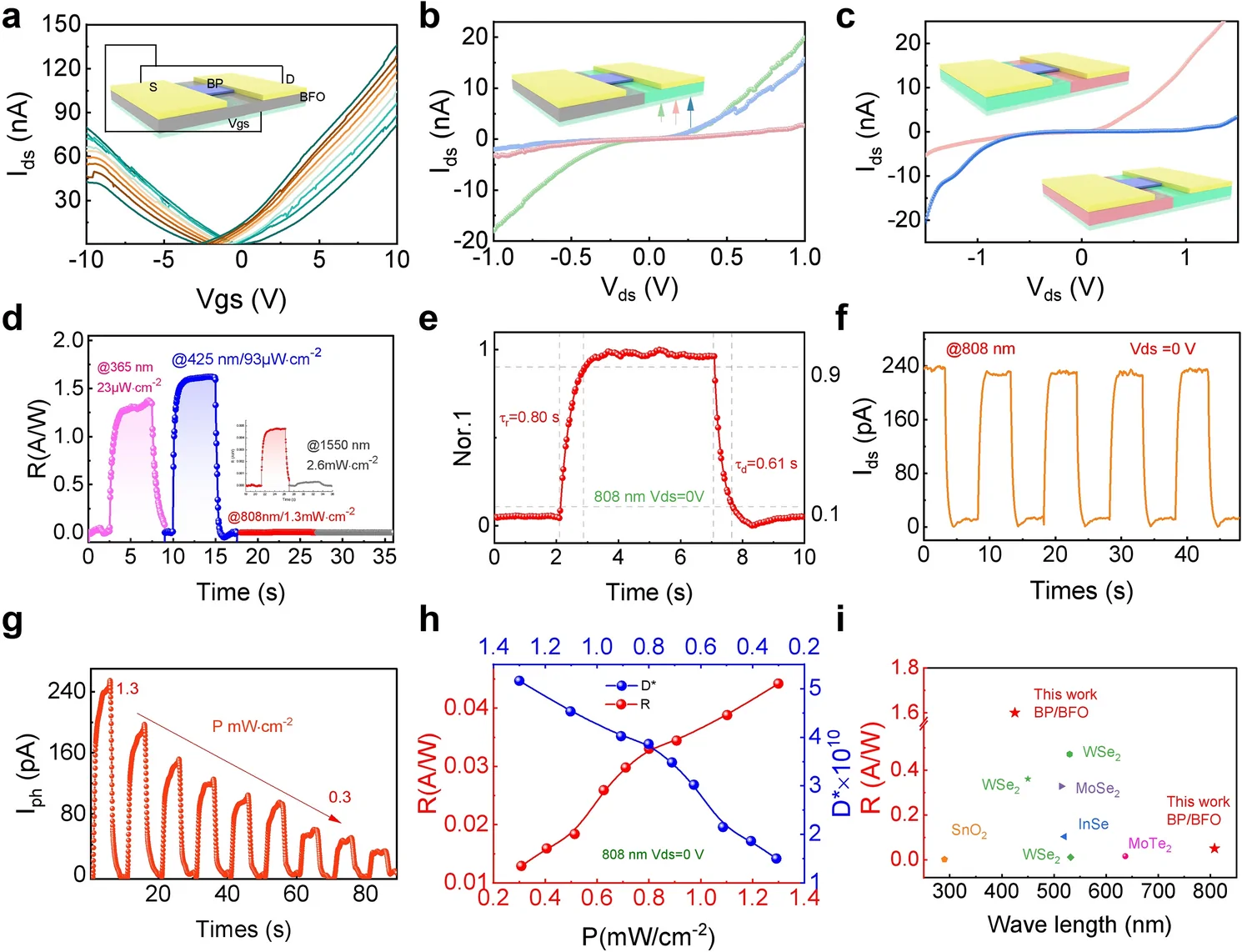
Optoelectrical characteristics of BP/BFO devices. **a** Transfer characteristic of a BP transistor. **b–c** Reversible transition from symmetric I–V behavior to rectifying p–n/n–p junctions under opposite ferroelectric polarizations. **d** Responsivity-calibrated photoresponse curves of the BP/BFO device under illumination at 365, 425, 808, and 1550 nm at 0 V bias. **e** Normalized transient photocurrent response under 808 nm illumination at 0 V bias. **f** Repeatable I–t response under 808 nm illumination at 0 V bias. **g** Photocurrent responses under different illumination powers at 808 nm at 0 V bias. **h** Power-dependent R and D* as functions of incident laser power density at 808 nm at 0 V bias. **i** Benchmark plots comparing the 0 V photoresponsivity with other self-powered photodetectors

The fabricated BP exhibits excellent intrinsic electrical properties, as confirmed by output (Fig. S5) and transfer characteristics of a BP bipolar transistor (Fig. 4a). The output characteristics indicate a symmetric gate voltage-modulation behavior. A reconfigurable p–n homojunction in a single BP nanosheet was achieved by selectively polarizing the underlying ferroelectric BFO domains (the polarization-writing configuration in Fig. S3c, d).Without polarization, the BP channel shows symmetric I–V behavior. Applying a polarization voltage (+ 5 V, + 10 V) to one electrode induces asymmetric, rectifying I–V characteristics (Fig. 4b). This occurs because the ferroelectric polarization locally controls carrier doping: a* P*_down_ domain p-dopes the BP, while a *P*_up_ domain n-dopes it. By placing the source and drain on oppositely polarized domains, we reversibly form either a p–n or n–p homojunction, as evidenced by their opposite rectification behaviors (Fig. 4c).

This ferroelectric gating also enables high-performance, self-powered photodetection. The BP/BFO device generates a broadband photoresponse from visible (365 nm) to infrared (1550 nm), with a peak responsivity at 425 nm (Fig. 4d). Under 808 nm illumination, the device exhibits rise and decay times of 0.80 and 0.61 s, respectively (Fig. 4e), and a stable, repeatable photocurrent (Figs. 4f and S6). The photocurrent scales with optical power, reaching 240 pA at the highest intensity (Fig. 4g). The dark current at 0 V bias remains stable over the measurement conditions and is 9.16 × 10^−12^ A, which is used consistently for the D ∗ calculations across the spectral range. Notably, the device achieves a self-powered responsivity (R) of 44 mA W^−1^ and a specific detectivity (D*) of 5.16 × 10^10^ Jones at 808 nm, and a remarkable 1.6 A W^−1^ and 1.8 × 10^12^ Jones at 425 nm (Figs. 4h and S7).

As shown in Fig. 4i and Table S2, the self-powered performance of our ferroelectric-gated BP/BFO device at 0 V bias is competitive with, and often superior to, most reported 2D material-based self-powered photodetectors [24, 31–36]. Furthermore, the photocurrent is tunable by the junction configuration, with the p–n state showing a higher photocurrent than the n–p state under identical illumination (Fig. S8). In summary, BFO's ferroelectric polarization not only creates reversible BP homojunctions but also provides a built-in electric field for efficient, broadband, self-powered photodetection.

### Differential Imaging

**Fig. 5:**
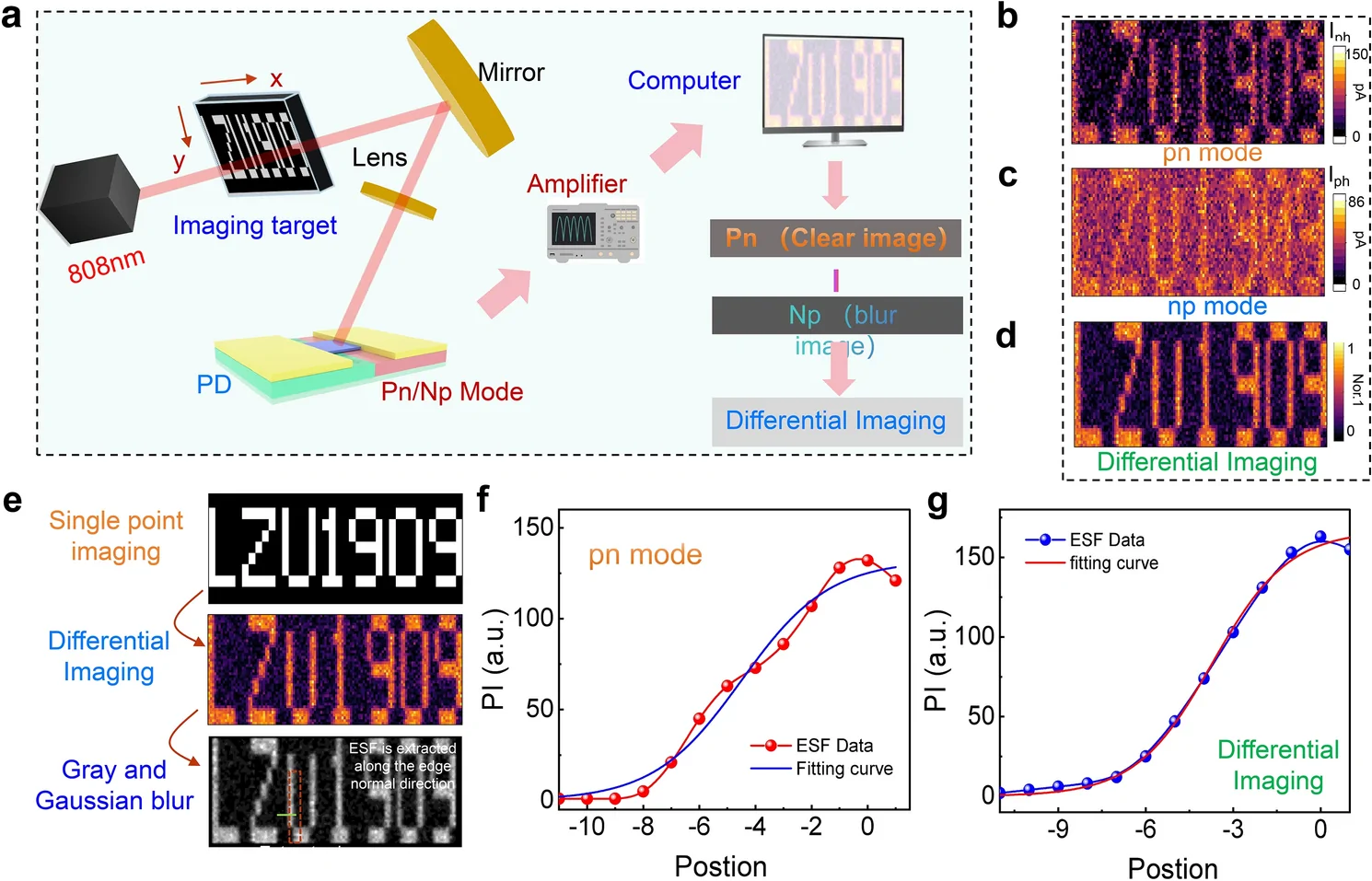
Differential imaging based on the BP homojunction device. **a** Schematic of the differential imaging scanning system using the BP homojunction device. **b, c** Single-pixel photocurrent imaging of the p–n and n–p configurations under 808 nm illumination at V_ds_ = 0 V and 1.3 mW·cm^−2^ power density. **d** Normalized differential image. **e** Photocurrent map generated when the laser passes through an “LZU1909” patterned mask, the corresponding differential image, and the edge-extracted result obtained by grayscale conversion, Gaussian blurring, and ESF fitting along the edge normal. **f** Local PI profiles extracted from the p–n scan and** g** the differential image, with their ESF fits

Leveraging the reversible reconfigurability of the BP/BFO heterojunction, a time–domain differential imaging strategy was proposed to suppress background noise and enhance image contrast, as illustrated in Fig. 5a. A laser beam passes through a moving mask patterned with the characters “LZU1909” before reaching the device, and the photocurrent generated at zero bias is used to reconstruct the image (0 V bias, 808 nm, 1.3 mW cm^−2^). By sequentially capturing images in the high-responsivity p–n state (*I*p–n) and the low-responsivity n–p state (*I*n–p), we can extract the differential signal *I*_diff_ =*|I*_*p–n*_* − I*_*n–p*_*|*. This operation effectively cancels out the common-mode static noise and dark current components that are polarization-independent. Consequently, the reconstructed differential image exhibits a significantly improved signal-to-noise ratio (SNR) and clearer edge definition compared to the individual states, demonstrating the potential for hardware-level image pre-processing (Fig. 5b–d).

To quantify the spatial resolution of the differential imaging approach, an edge sharpness analysis based on the edge spread function (ESF) was performed. The steepness of the ESF reflects the system’s ability to resolve edge transitions, with a steeper slope corresponding to sharper edges. The photocurrent image used for this analysis is shown in Fig. 5e. The raw image was converted to grayscale, smoothed by Gaussian filtering, and processed by the Canny operator to extract the edge boundary. Pixel intensity (PI) profiles were then sampled along the normal direction of the edge to construct the ESF curves (Fig. 5f, g). The ESF data (orange dots) were fitted with a sigmoid function (blue curve):

$$I\left(x\right)=\frac{{I}_{\mathrm{max}}}{1+{e}^{-k(x-{x}_{0})}}$$

where *x* denotes the coordinate along the edge normal, k represents the slope coefficient, *x*_*0*_ is the edge center position, and *I*_max_ is the maximum PI (0–255 for 8-bit images). For direct p–n imaging, the fitted ESF slope is *k* = 0.63 (Fig. 5f), whereas differential imaging increases the slope to *k* = 0.76 (Fig. 5g). The larger slope indicates a more abrupt edge transition, confirming that differential processing improves edge sharpness and effectively suppresses background interference.

## Conclusions

In summary, we have successfully demonstrated a reconfigurable BP p–n homojunction photodetector engineered via the non-volatile ferroelectric domain programming of a BFO substrate. This in situ ferroelectric doping strategy effectively circumvents the lattice damage and instability issues associated with conventional ion implantation or chemical doping, enabling the creation of high-quality, programmable rectifying junctions within a single device channel. The fabricated device exhibits excellent self-powered performance, achieving a responsivity of 44 mA W^−1^ and broadband detection capabilities from the visible to near-infrared regions without external bias. Furthermore, by leveraging the reversible switching between p–n and n–p configurations, we realized a single-pixel differential imaging prototype that significantly suppresses background noise and enhances edge definition. This work establishes a paradigm for ferroelectrically programmable 2D devices, providing a versatile platform for differential imaging and contrast-enhancement optoelectronic applications.

## Supplementary Information

Below is the link to the electronic supplementary material.

Supplementary file1 (DOCX 17827 KB)
