# Supplementary material for: High-Performance Differential Imaging via Reconfigurable Black Phosphorus p–n Homojunction Optoelectronics
Source: Nanomicro Lett. 2026 Feb 28;18:262. doi: 10.1007/s40820-026-02104-z (PMC12950119; doi:10.1007/s40820-026-02104-z)
Supplement: Supplementary file 1 — Supplementary file1 (DOCX 17827 KB) [file 40820_2026_2104_MOESM1_ESM.docx]

Supporting Informaiton for

**High Performance Differential Imaging via Reconfigurable Black Phosphorus p-n Homojunction Optoelectronics**

Rui Hao^1, 2^, Lili Luo^1^, Lu Yang^1^, Xue Yang^2^, Fengsong Gao^1^, Meijie Zhu^2^, Yingtao Li^1,^ *, Qingliang Feng^2,^ *, Zemin Zhang^1^*

^1^ School of Physical Science and Technology, Lanzhou University, Lanzhou 730000, P. R. China

^2^ School of Chemistry and Chemical Engineering, Northwestern Polytechnical University, Xi’an 710072, P. R. China

*Corresponding authors. E-mail: [ytli@lzu.edu.cn](mailto:ytli@lzu.edu.cn) (Yingtao Li); [fengql@nwpu.edu.cn](mailto:fengql@nwpu.edu.cn) (Qingliang Feng); [zhangzemin@lzu.edu.cn](mailto:zhangzemin@lzu.edu.cn) (Zemin Zhang)

**Supplementary Figures and Tables**

**Fig. S1** Schematic diagram of electrochemical exfoliation of BP (**a**) exfoliation BP nanosheets dispersed on the substrate (**b**)

**Fig. S2** The XRD pattern of BFO films

**Fig. S3** Schematic illustrations of polarization programming and characterization configurations. (**a**) Ferroelectric domain writing using a conductive AFM tip with the bottom LSMO electrode grounded. (**b**) PFM and KPFM measurements performed after removal of the DC writing bias, ensuring that the measured CPD reflects remanent polarization-induced surface potential. (**c**) Polarization programming via lithographically defined electrodes prior to device measurements. (**d**) Electrical and optoelectronic characterization of the programmed pn/np configurations without any external writing field

**Fig. S4** The Raman spectra of BFO films (**a**) and BFO/BP (**b**)

**Fig. S5** Output characteristics of the BP transistor device on BFO substrate

**Fig. S6** Repeatable I-t characteristics of the BP/BFO device under illumination at different wavelengths: 365 nm (**a**), 808 nm (**b**), 1550 nm


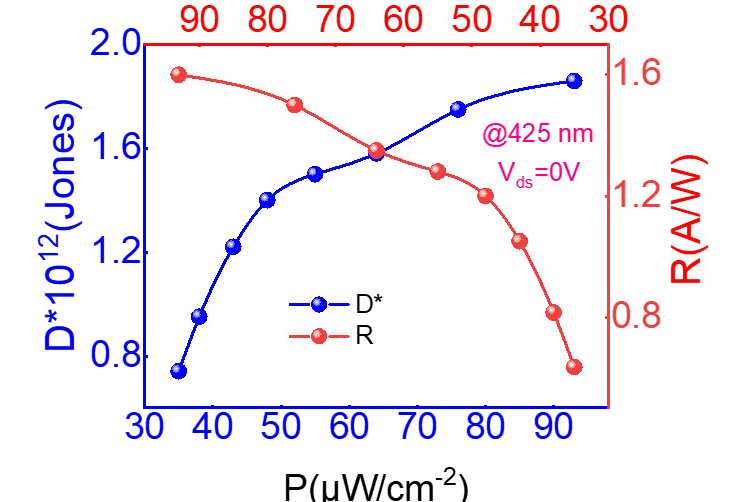


**Fig. S7** Power-dependent R and D* as functions of incident laser power density at 425 nm at 0 V bias

**Fig. S8** Photocurrent It curves under different p-n and n-p configurations

In this study, the carrier distribution of channeled BP nanosheets is statistically modeled using the Fermi-Dirac distribution, and the energy band structure of the material obtained by combining Poisson's equation, the current continuity equation, and the drift-diffusion model.Various physical effects are considered and the concentration distributions of electrons and holes are analyzed when different gate voltages are applied to different regions on the left and right, in order to determine the effect of voltage on the channel .The resulting TCAD simulation model matches the actual experimental junction.Some key models are briefly described below.

**SRH Model:**

SRH recombination refers to the recombination caused by the presence of localized energy levels or defects in semiconductors. The specific form of the SRH model is as follows:

$R_{SRH}=\frac{n_{0}p_{0}-n_{i}^{2}}{\tau_{n}\left( n_{0}+n_{1} \right)+\tau_{0}(p_{0}+p_{1})}$ (S1)

In the equation, R_SRH_ represents the recombination rate per unit volume, *n_0_​* and *p_0_* denote the electron and hole concentrations due to impurity defects, *n_1​_* and *p_1_* represent the intrinsic electron and hole concentrations of the semiconductor, *n_i_* is the intrinsic carrier concentration of the semiconductor, and *τ_n_* and τ_p_ are the lifetimes of electrons and holes, respectively.

**Carrier mobility dependency model**

The carrier mobility has a significant impact on the response speed of the photodetector. Due to the coexistence of multiple scattering mechanisms, the carrier mobility varies with doping concentration and electric field strength at room temperature. We use the built-in TCL language interpreter in Sentauras Sdevice to embed the corresponding model to account for this effect. For specific doping concentrations and electric field strengths, the corresponding carrier mobility can be calculated using the following formula:

$\mu_{h_{0}}\left( N_{imp} \right)=\mu_{min}+\frac{\mu_{max}-\mu_{min}}{1+{(\frac{N_{imp}}{N_{\mu}})}^{\alpha}}$ (S2)

$\mu_{h}\left( E,N_{imp} \right)=\mu_{h_{0}}(N_{imp})\left( \frac{1}{1+(\frac{\mu_{h_{0}}E_{f}}{\nu_{sat}})^{\beta_{sat}}} \right)^{1/\beta_{sat}}$ (S3)

Where $\mu_{h_{0}}$ and *μ_h_* are the hole mobility without electric field or with electric field, *N_imp_* is the doping concentration, *E_f_* is the electric field, ν_sat_ is the saturation velocity for the hole, and *a*, *μ_max_*, *μ_min_*, *N_m_*, *β_sat_* are the fitting parameters.

**FE Polarization model:**

The ferroelectric (FE) model describes the relationship between polarization and electric field in ferroelectric materials based on the Ginzburg–Landau–Khalatnikov (GLK) formalism. It characterizes the evolution of spontaneous polarization under an external electric field through minimization of the system’s free energy. The general form of the FE model is expressed as:

$\rho\frac{dP_{i}}{dt}+\nabla_{P_{i}}F=0$ (S4)

where P represents the polarization vector, ρ is a material-dependent kinetic coefficient, and F(P) denotes the free energy function expanded as a power series of P:

$F=\int_{\Omega} \alpha_{i}P_{i}^{2}+\beta_{i}P_{i}^{4}+\gamma_{i}p_{i}^{6}-g_{ij}\frac{\partial P_{i}}{\partial x_{j}}-E_{i}P_{i}+\varepsilon_{0}E_{i}E_{i}d\Omega$ (S5)

Here, α, β, and γ are material-specific parameters, and E is the applied electric field. The presence of multiple energy minima in F(P) leads to polarization switching between different states, resulting in ferroelectric hysteresis and remanent polarization. This behavior provides the physical foundation for nonvolatile electric-field control and underlies the operation of ferroelectric field-effect and negative-capacitance devices.

In summary, **Table S1** summarizes the main material parameters used in the aforementioned models.

**Table S1** Parameters adopted in TCAD simulation

| Parameter | BP | BFO |
| --- | --- | --- |
| Bandgap（eV） | 0.3 | 2.8 |
| Electron affinity（eV） | 4.0 | 4.0 |
| Relative permittivity | 8.4 | 85 |
| Shockley-Read-Hall |  |  |
| τ_n0_ | 1.0×10^-8^ | 1.0×10^-9^ |
| τ_p0_ | 1.0×10^-9^ | 1.0×10^-10^ |
| Mobility |  |  |
| μ_n_（cm^2^·V^-1^·s^-1^） | 1000 | 50 |
| μ_p_（cm^2^·V^-1^·s^-1^） | 800 | 20 |
| Vsn（cm·s^-1^） | 1×10^17^ | 1×10^18^ |
| Vsp（cm·s^-1^） | 5×10^5^ | \ |
| FE Polarization |  |  |
| Alpha | 0.0 | -2.5e+10 |
| Beta | 0.0 | 1.8e+20 |
| Gamma | 0.0 | 0.0 |
| G | 0.0 | 2.0e-03 |
| Rho | 0.0 | 1.0e+06 |

**Table S2** Benchmark comparison of self-powered photodetectors

| Device structure | 𝜆(nm) | R(A/W) | D (Jones*10^10^) | Pin(μW/cm^-2^) | τ_d_(ms) |
| --- | --- | --- | --- | --- | --- |
| This work | 425 | 1.6 | 12 | 93 | 600 |
| WS_2_ | 530 | 0.471 | - | 12300 | 4.4 |
| WSe_2_ | 450 | 0.36 | - | 6000 | 0.61 |
| SnO_2_ | 290 | 0.00155 | 9.8 | 18 | 0.64 |
| WSe_2_- WSe_2_ | 532 | 0.0112 | 4.40 | 500 | 0.18 |
| InSe | 520 | 0.103 | 1.83 | 54.4 | 0.103 |
| 1D Te/2D MoSe_2_ | 514 | 0.328 | 0.82 | 39000 | 0.328 |
| MoTe_2_ | 637 | 0.016 | 10 | 1.13 | 0.016 |

**Supplementaty References**

1. J. Park, Y. You, D. Lee, J. Kim, Self-powered high-performance WS_2_ photodetector *via* a monolithic p-i-n homojunction. Nano Lett. **25**(37), 13780–13786 (2025). <https://doi.org/10.1021/acs.nanolett.5c03066>
2. J. Weng, Z. Zhang, T. He, L. Zhang, Y. Zhou et al., WSe_2_ pn homojunction photodetector engineered by *in situ* ferroelectric doping. Adv. Funct. Mater. **35**(43), 2506469 (2025). <https://doi.org/10.1002/adfm.202506469>
3. B. Liu, M. Li, W. Fu, P. Ye, W. E et al., High-performance self-driven ultraviolet photodetector based on SnO_2_ p-n homojunction. Opt. Mater. **129**, 112571 (2022). <https://doi.org/10.1016/j.optmat.2022.112571>
4. C. Tan, H. Wang, X. Zhu, W. Gao, H. Li et al., A self-powered photovoltaic photodetector based on a lateral WSe_2_–WSe_2_ homojunction. ACS Appl. Mater. Interfaces **12**(40), 44934–44942 (2020). <https://doi.org/10.1021/acsami.0c11456>
5. J. Chen, Z. Zhang, J. Feng, X. Xie, A. Jian et al., 2D InSe self-powered Schottky photodetector with the same metal in asymmetric contacts. Adv. Mater. Interfaces **9**(35), 2200075 (2022). <https://doi.org/10.1002/admi.202200075>
6. J. You, Z. Jin, Y. Li, T. Kang, K. Zhang et al., Epitaxial growth of 1D Te/2D MoSe_2_ mixed-dimensional heterostructures for high-efficient self-powered photodetector. Adv. Funct. Mater. **34**(10), 2311134 (2024). <https://doi.org/10.1002/adfm.202311134>
7. K. Shao, H. Nan, R. Qi, C. Jiang, H. Wang et al., Self-powered MoTe_2_ homojunction photodetector with ultrafast response *via* h-BN encapsulation and doping regulation. J. Mater. Chem. C **13**(36), 18972–18980 (2025). <https://doi.org/10.1039/D5TC02340H>
